# Encapsulated vaterite-calcite CaCO_3_ particles loaded with Mg^2+^ and Cu^2+^ ions with sustained release promoting osteogenesis and angiogenesis

**DOI:** 10.3389/fbioe.2022.983988

**Published:** 2022-08-11

**Authors:** Lu Fan, Fabian Körte, Alexander Rudt, Ole Jung, Claus Burkhardt, Mike Barbeck, Xin Xiong

**Affiliations:** ^1^ NMI Natural and Medical Sciences Institute at the University of Tübingen, Reutlingen, Germany; ^2^ Experimental Medicine, Faculty of Medicine, University of Tübingen, Tübingen, Germany; ^3^ Faculty of Applied Chemistry, Reutlingen University, Reutlingen, Germany; ^4^ Medical Center of Rostock University, Rostock, Germany

**Keywords:** bioactive cations, vaterite-calcite, polyelectrolyte multilayer, collagen, bone regeneration

## Abstract

Bioactive cations, including calcium, copper and magnesium, have shown the potential to become the alternative to protein growth factor-based therapeutics for bone healing. Ion substitutions are less costly, more stable, and more effective at low concentrations. Although they have been shown to be effective in providing bone grafts with more biological functions, the precise control of ion release kinetics is still a challenge. Moreover, the synergistic effect of three or more metal ions on bone regeneration has rarely been studied. In this study, vaterite-calcite CaCO_3_ particles were loaded with copper (Cu^2+^) and magnesium (Mg^2+^). The polyelectrolyte multilayer (PEM) was deposited on CaCuMg-CO_3_ particles *via* layer-by-layer technique to further improve the stability and biocompatibility of the particles and to enable controlled release of multiple metal ions. The PEM coated microcapsules were successfully combined with collagen at the outmost layer, providing a further stimulating microenvironment for bone regeneration. The *in vitro* release studies showed remarkably stable release of Cu^2+^ in 2 months without initial burst release. Mg^2+^ was released in relatively low concentration in the first 7 days. Cell culture studies showed that CaCuMg-PEM-Col microcapsules stimulated cell proliferation, extracellular maturation and mineralization more effectively than blank control and other microcapsules without collagen adsorption (Ca-PEM, CaCu-PEM, CaMg-PEM, CaCuMg-PEM). In addition, the CaCuMg-PEM-Col microcapsules showed positive effects on osteogenesis and angiogenesis in gene expression studies. The results indicate that such a functional and controllable delivery system of multiple bioactive ions might be a safer, simpler and more efficient alternative of protein growth factor-based therapeutics for bone regeneration. It also provides an effective method for functionalizing bone grafts for bone tissue engineering.

## 1 Introduction

Various materials and functionalization strategies have been developed and extensively investigated in the field of bone regeneration (Lee et al., 2019; O'Neill et al., 2018). Nevertheless, effective vascularization, mineralization and tissue remodeling of regenerating bone tissue remains the main bottleneck for most graft-induced bone healing (Collins et al., 2021; Divband et al., 2021). A major research focus has been the incorporation of protein growth factors into bone implants to improve the efficacy of treatment. Such as the bone morphogenetic protein-2 (BMP-2) has been used clinically to promote bone healing since 2002 due to its diverse functions and osteogenic potential (Halloran et al., 2020; Hettiaratchi et al., 2020). Vascular endothelial growth factor (VEGF) is widely used in bone tissue engineering research for the improvement of tissue vascularization, either alone or in combination with BMP-2 (Liu et al., 2020; Fitzpatrick et al., 2021). However, significant disadvantages are associated with these protein growth factors-based therapeutics, including the sever complications caused by the supraphysiological dose applied, the instability in the fabrication process, and the high cost (Ruehle et al., 2019; Gelebart et al., 2022). Repeated clinical applications of BMP-2 have not been approved as safe and effective by the United States Food and Drug Administration (FDA) (SSED, 2022). Therefore, the alternatives of protein growth factor-based therapeutics are in demand.

Many bioactive metal ions have been shown to modulate osteoblast precursor differentiation *via* growth factor signaling pathways, or other processes to promote bone tissue regeneration (Glenske et al., 2018; O'Neill et al., 2018). Compared to the applications of protein growth factors, the advantages of using metal ions to induce bone tissue repair are manifold, including lower cost, greater simplicity, higher stability, and better efficacy at low concentrations (Glenske et al., 2018; Hurle et al., 2021). One of those efficient bioactive metal ions showed great importance in bone regeneration is calcium ion (Ca^2+^). The incorporation of Ca^2+^ into bone repair scaffolds has been shown to promote adhesion, proliferation, and differentiation of osteoblasts (Xie et al., 2018; Jeong et al., 2019). Copper ions (Cu^2+^) show great potential for vascularization, which are critical component of bone formation and tissue engineering (Lin et al., 2020; Kargozar et al., 2021). 50–60% of magnesium ions (Mg^2+^) are bound in bone and about 40% of Mg^2+^ are sorted in soft tissue (Musso, 2009). Many studies have shown increased bone growth around degradable Mg-alloys (He et al., 2016). Besides, Mg^2+^ showed positive influences to enhance matrix mineralization, osteogenic genes and protein expression of human bone marrow stem cells and osteoblasts (Qiao et al., 2021; Zhao et al., 2021). It should not be underestimated that bioactive cations are able to diffuse through the cellular membrane and regulate the activity of a variety of physiological responses (Mourino et al., 2012; Qiao et al., 2021). Thus, the metal ions induced concentration-dependent cytotoxicity and nonspecific adverse effects might be observed in neurological, cardiological, hematological, and/or endocrine systems (O'Neill et al., 2018). To reduce or avoid these side effects, a stable delivery system is required that can sustain ion release in the bone defect area both temporally and spatially. On this basis, it is of interest to investigate the synergetic therapy of multiple metal ions for bone regeneration.

Calcium carbonate in its vaterite form is widely used as a sacrificial template for efficient drug delivery. CaCO_3_ vaterite based delivery platforms have exhibited numerous advantages, such as the high loading efficiency, beneficial porosity, high mechanical stability, biodegradability preferential safety profile, simple preparation and low cost (Vikulina et al., 2021; Daria et al., 2022; Feng et al., 2022). However, the application as a platform for the delivery of proteins, and their long term and controlled release are limited, because the high ionic strength and pH changes during vaterite formation and sacrifice process would result in a dramatic loss of protein activity and a burst release of the encapsulated drug (Feoktistova et al., 2020). However, they do not impact CaCO_3_ being an ideal platform for the delivery of metal ions. Not only as a sacrificial template, CaCO_3_ is also one of the cargos, because it contains the important bioactive cations, Ca^2+^. It was reported that the CaCO_3_ particles could be converted into hydroxyapatite crystals in mild acidic environment (Wei et al., 2015). It enables the steady and continuous release of targets as it biodegrades *in vivo*. The CaCO_3_ crystals are particularly suitable for loading with multiple active pharmaceutical ingredients by a simple co-precipitation technique. Polyelectrolyte multilayer (PEM) deposited onto the degradable vaterite CaCO_3_ crystals *via* a layer-by-layer (LbL) process have served as multifunctional and tailored vehicles for advanced drug delivery (Campbell et al., 2020). Due to their tunable and inherent properties, PEMs can further enhance the biodegradability and bioactivity of CaCO_3_ crystals, and efficiently inhibit the initial burst release of cargos caused by the complex microenvironment *in vivo*.

**FIGURE 1:**
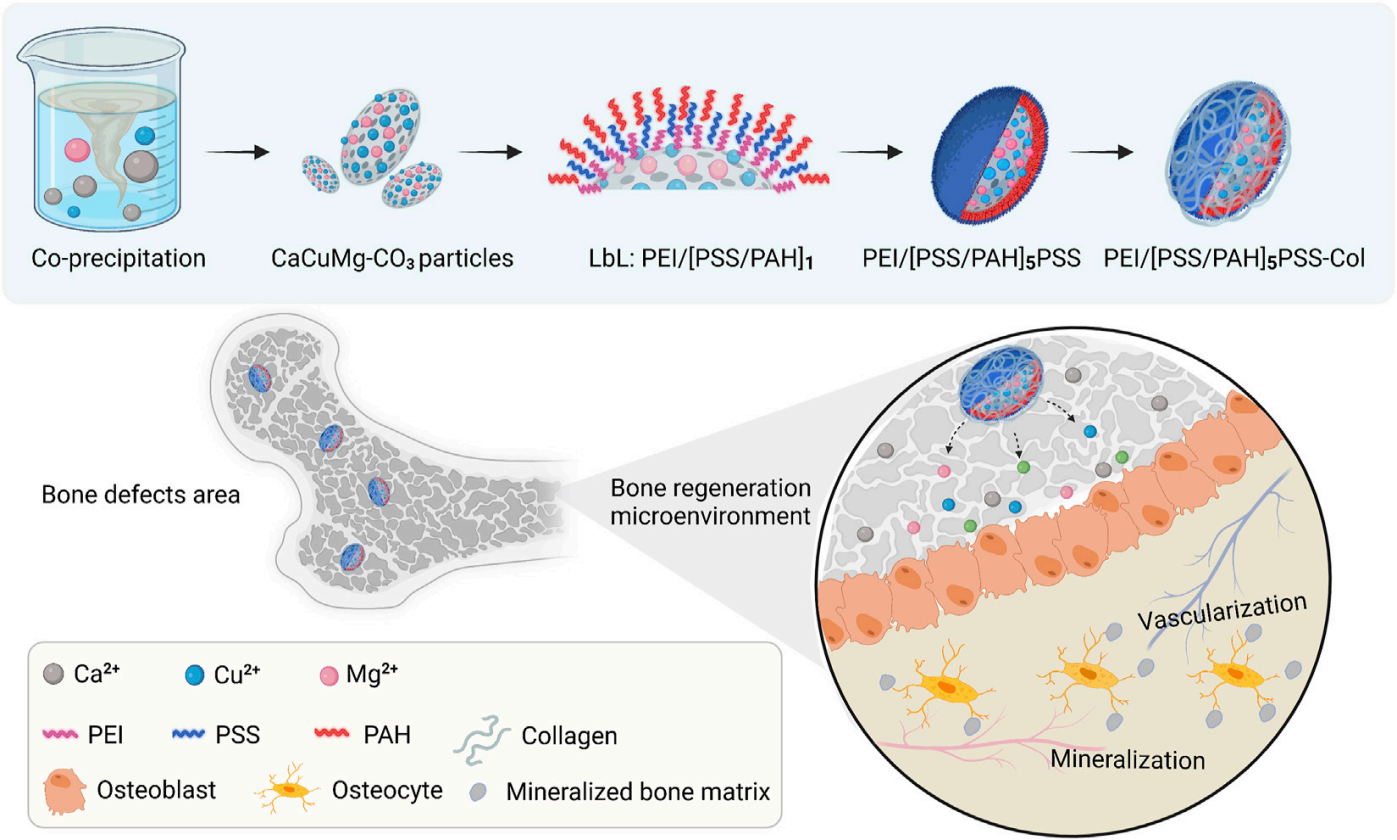
Schematic illustration of the fabrication process of CaCuMg-PEM-Col microcapsules [CaCuMg-CO_3_ particles + PEI/(PSS/PAH)_5_PSS + Col], and their effects on bone regeneration.

Collagen is the main organic component in the bone matrix. It plays a crucial role in the bone formation and remodeling process (Toosi & Behravan, 2020). It has been widely reported that the incorporation of collagen can significantly improve the mechanical properties, osteoinductivity and osteogenicity of scaffold materials for bone tissue engineering (Zhang et al., 2018; Yu et al., 2020; Zhong et al., 2022). In many cases, due to the formability, homogeneity and reproducibility, coating is a preferable choice to integrate collagen into different bone matrices and scaffolds as well as inorganic bone substitute materials. Brito Barrera et al. fabricated an osteogenic microenvironment relying on PEMs in combination with multilayers of type I collagen and chondroitin sulfate (Brito Barrera et al., 2020). The LbL technique is a method to fabricate coatings by alternate adsorption of polyanions and polycations, which can neatly combine collagen and PEM (Martin et al., 2021). In this study, the CaCO_3_ particles were loaded with Cu^2+^ and Mg^2+^ *via* a co-precipitation process. The co-precipitated particles were further encapsulated with PEMs and collagen, aiming for an elongated and constant release of bioactive cations. Simultaneously, the biofunctionalization of particle surfaces by PEMs and collagen simulated the extracellular microenvironment, together with released bioactive ions promoting bone tissue regeneration. (Figure 1)

## 2 Materials and methods

### 2.1 Preparation and characterization of polyelectrolyte multilayers

The polyethyleneimine (PEI, Mw 750 kDa, 50 wt% in water), poly (sodium 4-styrene-sulfonate) (PSS, Mw 70 kDa) were purchased from Sigma-Aldrich, Germany. Poly (allylamine hydrochloride) (PAH, 120–200 kDa) was purchased from Alfa Aesar, Germany. Sodium chloride (NaCl > 99%) was purchased from Sigma-Aldrich, Germany. Collagen from porcine skin (collagen type I content > 90%) was provided by Biotrics bioimplants AG, Germany. Acidic acid (HAc) was purchased from Carl Roth, Germany. All materials were used without further purification. The deionized water (ddH_2_O) used in all experiments was prepared in a three stage Milli-Q plus purification system and had a resistivity higher than 18.2 MΩ cm^−1^.

The polyelectrolytes (PEs) solutions were prepared as follows: PEI was dissolved in ddH_2_O at a concentration of 0.01 monomer mol·L^−1^, pH ∼7.0. PSS and PAH were respectively dissolved in 0.5 M of NaCl at a concentration of 2 mg ml^−1^ and adjusted to pH 7.0. Collagen was dissolved in 0.1 M HAc at a concentration of 2 mg ml^−1^ at 4°C overnight under continuous agitation at 60 rpm and thereafter the pH was adjusted to pH 5.0 using NaOH.

The deposition was performed manually using the layer-by-layer (LbL) technique as reported previously (Sun et al., 2017). Gold-crystal sensors (QSX 301 Gold, Biolin scientific, Sweden) were used as a model surface. To characterize the buildup process and the properties of the deposited PEMs, quartz-crystal microbalance with dissipation monitoring (QCM-D) measurement using a Q-Sense E4 instrument (Biolin scientific, Sweden) was employed. Firstly, the QCM sensors were treated with Piranha solution (30% (v/v) H_2_O_2_, 70% (v/v) H_2_SO_4_) and washed extensively with ddH_2_O. PEI was always applied as precursor layer with a positive charge on the substrate followed by repeated rinsing in ddH_2_O (three times for 2 min each). After the deposition of PEI, alternate polyanionic PSS and polycationic PAH layers were deposited up to the desired amount of layers [PEI(PSS/PAH)_5_ or PEI(PSS/PAH)_5_PSS] at pH 7.0 with 10 min incubation for each layer. Each adsorption step was followed by three consecutive rinsing steps with ddH_2_O for 2 min each. Prior to the collagen deposition, the PEM layers were equilibrated in pH 5.0 ddH_2_O. Collagen was deposited as the last layer by incubating the sensors in collagen solution for another 10 min. The PEM and collagen buildup process was performed at 22°C. Finally, sensors were immersed in ddH_2_O overnight at 37°C for the stability measurements. In brief, the buildup process for the following systems have been monitored on QCM-D: 1) PEI[PSS/PAH]_5_ at pH 7.0 switch to pH 5.0 and followed by addition of collagen at pH 5.0; 2) PEI[PSS/PAH]_5_PSS at pH 7.0 switch to pH 5.0 and followed by addition of collagen.

The increase in mass adsorbed on the surface of the quartz crystal sensor leads to a decrease in the oscillation frequency.(Marx, 2003) The frequency shift (−Δ*f*) measured after the deposition of each polyelectrolyte provides direct evidence of adsorption of polyelectrolytes (Aggarwal et al., 2013). The mass of adsorbed polyelectrolyte (Δ*m*) can be calculated from the frequency shift using the Sauerbrey equation:

$$\Delta m=-c\frac{\Delta f}{n}$$

where *n* (*n* = 1, 3, and 5... 13) is the overtone number and *c* is the mass sensitivity constant given by the property of the used quartz crystal (Sauerbrey, 1959). In the present study, *f*_0_ is 5 MHz and *c* is17.7 ng Hz^−1^ cm^−2^ as stated by the manufacturer. The adsorbed mass and thickness of the PEMs were calculated using the D-Finder program according to the manufacturer’s instruction (Biolin scientific, Sweden). All QCM-D measurements were performed using “open module” system.

### 2.2 Fabrication of Cu^2+^ and Mg^2+^ loaded vaterite CaCO_3_ (CaCuMg-CO_3_) particles

Calcium carbonate particles loaded with Cu^2+^ and Mg^2+^ were fabricated on the basis of the previously established co-precipitation approach with some modifications (Sun et al., 2017). Briefly, 0.33 M chloride salt solutions composed of CaCl_2_, CuCl_2_ and MgCl_2_ (Sigma-Aldrich, Germany) at the ratio of *1*) 90%: 5%: 5%, *2*) 80%: 10%: 10%, *3*) 60%: 20%: 20%, *4*) 40%: 30%: *30%* were prepared and stirred at 500 rpm in 50 ml falcon tubes. 0.33 M Na_2_CO_3_ (Sigma-Aldrich, Germany) solutions with equal volumes were dropped into the respective chloride salt solutions under continuous stirring at 500 rpm with constant speed (0.6 ml/min) using a syringe pump. Stirring was continued for 1 min after the addition of the Na_2_CO_3_ solution. The obtained slurries were allowed to stand for 10 min and then centrifuged at 2,719 *g* for 2 min. The pellets were quickly resuspended in ddH_2_O and centrifuged again for the removal of excessive salts from the carbonates. Afterwards the carbonates were washed three times. The full procedure was performed at 22 ± 1°C.

### 2.3 Encapsulation with polyelectrolyte multilayers and collagen

To enable the desired sustained release and to improve the biocompatibility, the above-described PEM system with collagen as the most-outer layer was applied for the encapsulation of CaCuMg-CO_3_ particles. The freshly prepared CaCuMg-CO_3_ particles were encapsulated with PEI[PSS/PAH]_5_/PSS-Col in the same manner as described in 2.1 and 2.2. After each deposition and washing step, the particles were quickly but fully resuspended. The incubation with the respective PEs or collagen were carried out using a tube roller at room temperature. Similarly, each deposition step was followed by centrifugation (2,719 *g*, 2 min) and repeated washing with ddH_2_O for three times. The obtained CaCuMg-PEM-Col capsules were lyophilized using a Christ-ε 1-4 LSC plus device (Martin Christ, Osterode am Harz, Germany) and stored at room temperature for subsequent analysis.

For the further investigation of synergistic effects of Cu^2+^, Mg^2+^ and collagen, *1*) Ca-PEM, *2*) CaCu-PEM, *3*) CaMg-PEM; *4*) CaCuMg-PEM; *5*) CaCuMg-PEM-Col capsules were prepared according to the previously described methods. The ratio of each metal salt was *40%: 30%: 30%*.

### 2.4 Characterization of CaCuMg-PEM-Col capsules

#### 2.4.1 ζ-potential measurements

The charge and compensation of the charge by alternating adsorption of the polyelectrolytes and collagen are closely related to the properties of the PEM, collagen and the capsules. To monitor the charge compensation and characterize the surface, the ζ-potentials of the surface during the LbL-encapsulation was tracked. The electrophoretic mobility of the microcapsules was measured by photon correlation spectroscopy using a Zetasizer NanoZS (Malvern, Herrenberg, Germany). All measurements were performed at 25°C. The mobility was converted into a ζ-potential using the Smoluchowski relation and the system algorithm provided by Malvern (Salmivirta et al., 1996). Immediately after each layering-washing-resuspension process, surface charge was characterized by the ζ –potential (*n* = 3). Samples were always fully resuspended in ddH_2_O prior to the measurements. The measurements were performed in triplicate at each adsorption step. Samples were always fully dispersed in distilled water in equilibrium with the room atmosphere. 1 mmol L^−1^ KCl solution was applied as the model electrolyte, and 0.1 mol L^−1^ NaOH was used for pH titration from pH 3.0 to 10.0.

#### 2.4.2 Scanning electron microscopy and energy dispersive X-Ray analysis

Scanning electron microscopy (SEM) images and energy dispersive X-Ray analysis (EDS) of capsules were performed with a FIB/SEM microscope (Zeiss crossbeam 550) equipped with an EDS detector (Oxford X-Max). With this set-up we characterized the shape, size, surface morphology and element composition of CaCuMg-PEM-Col capsules. For this observation the lyophilized samples were attached firmly to carbon tape on a SEM stub without sputter coating. SEM images were then taken at accelerating voltages between 1.5 and 3 kV. The EDS analysis was performed with SEM at an acceleration voltage of 8 kV. The EDS analysis clearly show the distribution of Magnesium, Copper and Calcium in the samples.

#### 2.4.3 Fluorescence microscopy

For the visualization of assembled PEM and adsorbed collagen, fluorescence microscopy (Keyence BZ-X800, KEYENCE Deutschland GmbH, Neu-Isenburg, Germany) was applied. FITC-labeled PAH and type I collagen (Biomol GmbH, Germany) were added to the respective unlabeled PAH and type I collagen solutions at a ratio of 4%. The coating procedure was the same as described above under exclusion of light. Two FITC labeled samples were prepared as *1*) CaCuMg-PEM_FITC-Col (FITC labeled PAH at 3rd bilayer) and *2*) CaCuMg-PEM-Col_FITC (FITC labeled collagen at the last layer). The samples were resuspended with ddH_2_O in a 96-well plate and observed directly under the fluorescence microscope.

### 2.5 Release kinetics of Ca^2+^, Cu^2+^ and Mg^2+^

The Ca^2+^, Cu^2+^ and Mg^2+^ release profiles were analyzed with a SPECTROBLUE ICP-OES (Inductive coupled plasma-optical emission spectroscopy) analyzer (SPECTRO/AMETEK, Kleve, Germany). Briefly, 0.11 g of each lyophilized sample was immersed in 11 ml Eagle’s minimal essential medium (MEM, Gibco) and incubated at 37°C under continuous agitation at 60 rpm. 10 ml supernatant was collected by centrifugation (2,719 *g*, 2 min) at the specified time points (10 min, 1, 3, 7, 14, 21, 28 and 60 days). After sampling, 10 ml of fresh MEM was added to the residual suspension to continue the release kinetics. For measuring the total ion content, the powder was immersed in 11 ml 5% HAc for 24 h (37°C, 60 rpm), then analyzed using ICP-OES. All solutions used were sterile filtrated using a 0.45 µm membrane filter and the samples were handled and kept sterile. After processing of the measurement signals by the instrument, the measured intensities of the elements were evaluated *via* the Smart Analyzer software.

### 2.6 Cell culture

In this study, the murine fibroblast cell line L929 (ATCC, CCL1) and human derived osteoblast-like MG63 (ATCC, CRL 142) osteosarcoma cells were purchased from ATCC (VA, United States). L929 cells were grown in RPMI medium 1,640 GlutaMAX supplemented with 10% (v/v) fetal bovine serum (FBS) and 1% penicillin/streptomycin (P/S). MG63 cells were cultivated with MEM plus supplemented with 10% FBS, 1% P/S, and 1% sodium pyruvate, 1% nonessential amino acids (NEAA) and 1% l-glutamine. All these materials were purchased from Gibco (Carlsbad, CA, United States). Both cells were grown in a humidified 5% CO_2_/95% atmosphere and passaged when cell confluence rate was over 80%.

### 2.7 Cell viability test by cell count kit-8

The cytotoxicity of the CaCuMg-PEM-Col capsules was evaluated by an extraction test and CCK-8 assay of L929 cells according to ISO 10993 part 5 (ISO 10993-5:2009, confirmed in 2017) (ISO, 2009). 10 mg ml^−1^ of CaCuMg-PEM-Col capsules, positive control (PC, polyurethane film containing 0.1% zinc diethyldithiocarbamate (ZDEC), RM-A, HatanoResearch Institute, Japan), negative control (high density polyethylene film, RM-C, HatanoResearch Institute, Japan) in L929 cell culture medium and blank (only cell culture medium) were incubated at 37°C with gentle shaking at 60 rpm for 24 h. The cells were seeded in 96-well plates (10^4^ cells/well) and cultured at 37°C for 24 h with 5% CO_2_. Then, the medium was replaced with the extracts. After culturing for further 24 h, the cell viability of L929 was determined by CCK-8 assay (Sigma-Aldrich, Germany). Briefly, the medium was thoroughly removed, and the cells were then incubated with fresh medium supplemented with 10% CCK-8 reagent. After incubation at 37°C for 2 h, the color change was determined by measuring the absorbance at 450 nm using a microplate reader (TECAN RAINBOW, Germany).

The MG63 cell viability was also evaluated using CCK-8 method. Cells were seeded in 96-well plates (10^4^ cells/well) cultured with complete medium for 24 h. Then, the medium was thoroughly aspirated and replaced by fresh culture medium supplemented with the extracts of capsules. After incubation for the designated times, the cell viability was measured using the CCK-8-assay. The medium was replaced every 3 days.

### 2.8 Alkaline phosphatase activity measurements

As a key osteogenic maker of osteoblastic differentiation, alkaline phosphatase (ALP) activity was determined by conversion of *p*-nitrophenyl phosphate (pNPP) into *p*-nitrophenol (pNP). Incubating MG63 cells with substrate solution (1 mg ml^−1^ pNPP, 50 mM glycine, 1 mM MgCl_2_, 100 mM TRIS, pH 10.5) at 37°C for 30 min, and then measuring the absorbance at 405 nm using a microplate reader (TECAN RAINBOW, Germany).

### 2.9 Relative gene expressions

Total RNA extraction, reverse-transcription and real-time PCR were performed by using the Cells-to-CT^TM^ 1-Step TaqMan^™^ Kit (Thermo Fisher Scientific, Rockford, IL, United States), conducted on a one-step PCR instrument (Applied Biosystems, Foster City, CA, United States) according to the manufacturer’s instruction. For analysis of osteogenic-related genes (COL1A1, MMP1, ALPL, BGLAP, RUNX2) and angiogenic-related genes (VEGFA and HIF1A), the relative expression of mRNAs was calculated using glyceradehyde-3-phosphate dehydrogenase (GAPDH) as an internal reference standard gene by the 2^−∆∆Ct^ method. All primers were purchased from Thermo Fisher Scientific, and more details are listed in Supplementary Table S1, Supporting information.

### 2.10 Alizarin red staining

The formation of mineralized matrix nodules after 14 days was determined by Alizarin Red staining. Briefly, MG63 cells were fixed with 99% ethanol at −20°C for 2 h, then washed with tape water for three times. After incubating with 0.5% Alizarin Red solution (pH 4.0, Carl Roth) for 30 min at room temperature and another three washing steps, the resulting staining was assessed with optical microscopy. The bound Alizarin Red was resolved with 10% Cetylpyridium chloride solution (Carl Roth) for photometric quantification at the absorbance of 562 nm using a microplate reader (TECAN RAINBOW, Germany).

## 3 Results and discussion

### 3.1 Optimization and characterization of PEM and collagen coating

**FIGURE 2:**
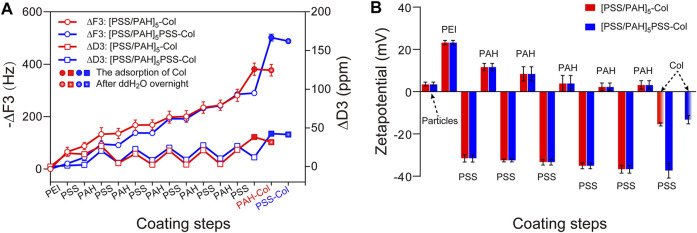
**(A)** QCM-D frequency (∆F, -o-) and dissipation (∆D3, -□-) plot for LbL coating process of PEM and collagen. The red broken line represents the buildup process of [PSS/PAH]_5_, further adsorption of collagen and incubation in ddH_2_O overnight; the blue broken line represents the coating steps of [PSS/PAH]_5_PSS-Col and further incubation in ddH_2_O overnight. **(B)** Zeta-potential measurement of respective surface charges during the PEM and collagen coating process on the basis of CaCuMg-CO_3_ crystals.

The buildup process of the PEM-systems was monitored by measurements using QCM-D (Figure 2A). The regular frequency shifts confirmed the effective adsorption of each deposited polyelectrolyte-layers and the assembly of the multilayer systems. The collagen was adsorbed on both basic layer-systems [PSS/PAH]_5_ and [PSS/PAH]_5_PSS with an average frequency shift of *−380.75 ± 23.45* *Hz* and *-501.77 ± 13.33* *Hz*. After overnight incubation in ddH_2_O only a slight increase of the frequency could be observed indicating a stable adsorption as reported previously. (Sun et al., 2017) More (113.23%) collagen could be adsorbed on top of the PSS (*212.06 ± 33.06* *Ηz*) than PAH (*99.45 ± 13.47* *Hz*). At a pH range from 4 to 10, collagen has both positive and negative charges throughout the molecules (Morozova and Muthukumar, 2018). The higher the pH value, the higher the negative charge measured. At a pH of 5, the collagen was predominantly present as positively charged molecules, resulting in greater adsorption on the negatively charged PSS surface compared to the positively charged PAH surface. After overnight incubation in ddH_2_O, the Δ*f* was 3.48 Hz (3.5% frequency increase) and 13.66 Hz (6.4% frequency increase) for PAH-Col and PSS-Col respectively. Energy dissipation was also monitored and plotted in Figure 2A. Δ*D* indicated primarily the change of the stiffness of the layers. Both PAH-Col and PSS-Col have reduction of dissipation and the Δ*D* was −6.6 ± 4.17 ppm and −1.14 ± 2.45 ppm respectively. Taking the results of Δf and Δ*D* together, it might be concluded that the PAH-Col and PSS-Col coatings retained their integrity after deposition because the negligible changes in frequency and mass. The increase in frequency is most likely due to the rearrangement of the PEM and collagen on the surfaces during the long time incubation in ddH_2_O. Also, the evaporation of the ddH_2_O in the used open cells could result in the slight fluctuation of the measurement. In addition, the fitting of the thickness using the QSense_Dfinder software showed a slight reduction of the thickness of the coatings indicating the tightening of the film-structure (Supplementary Table S1). After deposition and during equilibrium in water, PE molecules could complete the arrangement such as interpenetration due to compensation and stabilization of weak interactions, especially hydrogen-bonding and hydrophobicity-interactions between molecules (Wang et al., 2014). The small Δ*D* suggested a stable system has been formed and kept the stability during further incubation. The slight negative Δ*D* also indicates the increased stiffness and rigidity of the surface. As described in the literature, the lower overtone represents the adsorption and change at the interface between liquid and thin film, and the comparison of other signals recorded for higher overtones indicates the depth integrity of the system. The signals of overtones 3, 5 and 9 did not differ significantly from each other confirming the linearity, rigidity and rather stiffer property of the systems (Wang et al., 2014). As reported previously, molecular water can be pushed out while the structure of the film was rearranged to a much thinner and stable form. This process will also result in a slight frequency increase due to loss of water molecules from the film (Delajon et al., 2009; Kohler et al., 2009).

As mentioned above, collagen has both positively and negatively charged moieties, which allows adsorption to differently charged surfaces. The adsorption determined by QCM might be resulted from the adhesive property of collagen. ς-Potential confirmed the buildup process according to the charge-compensation theory of the PEM-LbL technique (Figure 2B). Moreover, the initial positive charge of the PAH outer layer was reduced to a negative charge (−18.67 mV) after adsorption of collagen, while the strong negative charge of PSS was strongly neutralized to (−24.10 mV) by collagen. This observation was in accordance with the expectation that collagen can be stably adsorbed both on positively and negatively charged surfaces at proper pH value. In one word, the buildup of the designed PEM systems was successfully performed and confirmed by QCM-D analysis. The stability of both systems was proven to be sufficient for further applications. The –PSS showed overall a better adsorption capacity of collagen and stability in comparison to –PAH.

### 3.2 Characterization of CaCuMg-PEM-Col microcapsules

**FIGURE 3:**
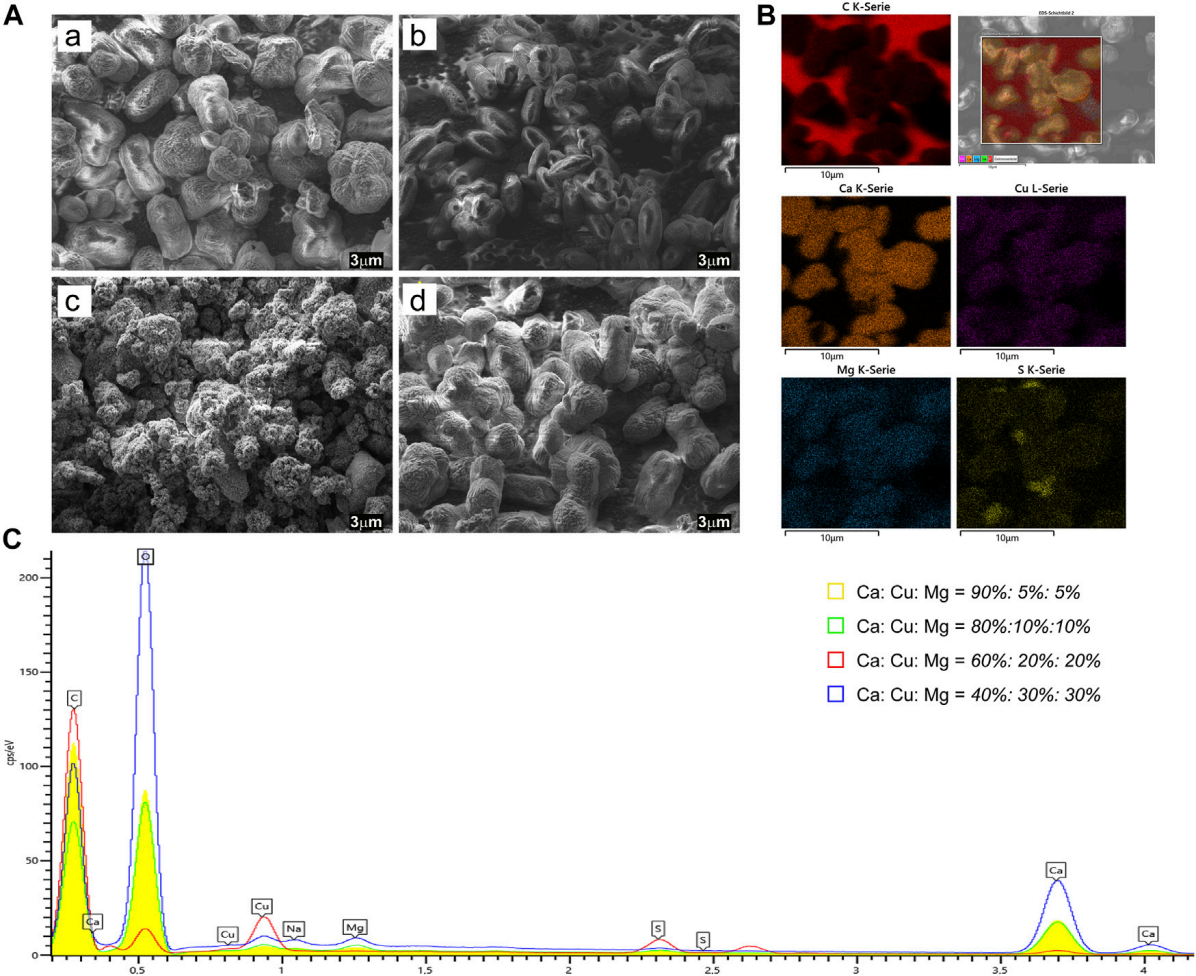
**(A)** SEM images of CaCuMg-PEM-Col microcapsules prepared with different ratios of salts: (a) Ca^2+^: Cu^2+^: Mg^2+^ = *90%: 5%: 5%*, (b) Ca^2+^: Cu^2+^: Mg^2+^ = *80%: 10%: 10%*, (c) Ca^2+^: Cu^2+^: Mg^2+^ = *60%: 20%: 20%*, (d) Ca^2+^: Cu^2+^: Mg^2+^ = *40%: 30%: 30%* (scale bar = 3 μm). **(B)** *C, Ca, Cu, Mg, S* element EDS-Mapping of microcapsules (the group of *40%: 30%: 30%*). **(C)** EDS spectra of CaCuMg-PEM-Col microcapsules prepared with four ratios of salts.

As mentioned above, the vaterite formation process of CaCO_3_ is easily influenced by the experimental conditions and the substances introduced during co-precipitation. In order to guarantee stable encapsulation and sustainable release with the desired controllability, different microcapsule formulations were prepared by co-precipitation. The morphology and elemental composition of CaCuMg-PEM-Col particles were investigated *via* SEM combined with EDS-analysis. When the ratio of Ca^2+^, Cu^2+^ and Mg^2+^ was 90%: 5%: 5%, the formed particles presented three kinds of morphologies: bigger spherical particles (41.38% of total) with an average size around 5.0 ± 0.5 μm (diameter), smaller spherical particles (20.69%) in the size of 2.6 ± 0.3 μm, and oval particles (37.93%, 5.6 ± 0.3 μm × 2.9 ± 0.3 μm) (Figure 3Aa [if subparts are of A]). The SEM image of the 80%: 10%: 10% group showed that most of the particles have an oval shape with relatively uniform size (Length 3.9 ± 0.4 μm Width1.9 ± 0.3 μm) (Figure 3Ab [if subparts are of A]). Interestingly, in the group of *60%: 20%: 20%*, there were no regular shaped vaterite or calcite particles observed. SEM images revealed irregular and porous structures (Figure 3Ac [if subparts are of A]). The particles obtained from the group of *40%: 30%: 30%* showed a similar morphology as the *90%: 5%: 5%* group. The bigger spherical particles in this sample were around 5.5 ± 0.4 μm and account for 12.90%; the smaller ones were in the size of 2.5 ± 0.4 μm (48.39%) and the oval particles were about L 5.474 ± 0.5 μm W 3.0 ± 0.4 μm (29.27%) (Figure 3Ad [if subparts are of A]).

As one of the most effective platforms for drug encapsulation and delivery, the key parameters for size- and shape-controlled synthesis of CaCO_3_ particles have been well studied. The fast mixing of aqueous calcium chloride and sodium carbonate can immediately result in amorphous calcium carbonate (ACC). Under vigorous stirring, the formed ACC in the precipitation system will dissolve first, and then transform within minutes to produce crystalline forms of vaterite and calcite (Shen et al., 2006). However, when the system is introduced with other divalent cations, the co-precipitation reaction and co-crystallization process will be more complicated, thus rarely being reported. In the co-precipitation system of Ca^2+^, Cu^2+^, and Mg^2+^ presented in this work, there are two main factors affecting the morphology of obtained particles: the concentration of Ca^2+^, and the ionic interactions with Cu^2+^ and Mg^2+^. It is known that the higher content of Ca^2+^ within a certain range would lead to high yield of vaterite; trace amounts of Cu^2+^, and Mg^2+^, which have a smaller ion size than Ca^2+^ could enter and distort the lattice of just formed CaCO_3_-crystals, and thus influence the size and morphology of the final particles, as shown in Figure 3Aa [if subparts are of A]) (Svenskaya et al., 2018). A certain proportion of Mg^2+^ was reported to promote the formation of vaterite CaCO_3_ crystals (Kulp and Switzer, 2007). Among the four ratios in this study, 10% of Mg^2+^ might play the optimal role in vaterite formation. Particles obtained at the ratio of *80%: 10%: 10%* showed the most regular oval vaterite morphology (Figure 3Ab [if subparts are of A]). With the increase of Cu^2+^ and Mg^2+^ content, the crystals gradually transformed from vaterite to calcite leading to a reduced uniformity in the particles. The particles from the ratio of 60%: 20%: 20% appeared to stay at the transformation phase of ACC-vaterite, and particles of 40%: 30%: 30% were at vaterite-calcite stage or mixture of different crystallization products (Figures 3Ac,d [if subparts are of A]). Most likely the ions find an equilibrium among vaterite and calcite during particle formation depending on the ratios and precipitation conditions. This could be due to the excessive electronegativity of Cu^2+^ and Mg^2+^, which led to a distortion of the crystal plane structure; furthermore, the increasing cocrystallization with CuCO_3_ (Cu_2_CO_3_(OH)_2_) and MgCO_3_ suppressed the formation and stability of CaCO_3_ vaterite. Additional influence from the PEM coating should also be considered as a morphology-stabilizing factor. The particles were encapsulated in their precipitated shapes without further modifications as a substrate for coating deposition. CaCO_3_ has a water solubility of 15 mg L^−1^ and MgCO_3_ 0.14 g L^−1^. More complicated, the Cu^2+^ will form so called basic copper carbonate consisting of Cu_2_CO_3_(OH)_2_ which has a negligible solubility in water. The basic copper carbonate has a typical monoclinic crystal and spertiniite morphology. Also the formation of dolomite, CaMg(CO_3_)_2_ would further enhance the alternating morphology due to the structural arrangement of the Ca^2+^ and Mg^2+^ ions. These properties derived from basic copper carbonate and dolomite could explain the morphological changes from ACC, to vaterite and calcite in this complex mixture system as shown in Figure 3A. These mixtures of dolomite and basic copper carbonate will also further affect the release kinetics by means of dissolution and degradation.

X-ray energy dispersive spectroscopic (EDS)-mapping images and spectra further revealed the element composition of CaCuMg-PEM-Col capsules. The SEM EDS-mapping images showed that the elements including C, Ca, Cu, Mg, S were uniformly distributed on particles prepared with these four ratios (Figure 3B and Supplementary Figure S1). The EDS spectra exhibited the characteristic peaks for C, Ca, Cu, Mg, and S (Figure 3C) present in the surfaces of the microcapsules. The obtained element composition ratio is shown in Supplementary Table S2. No significant difference in the element composition ratio of the group of 95%: 5%: 5% and 80%: 10%: 10% was observed. The high ratio of Cu and low ratio of Ca in the group of 60%: 20%: 20% suggested a high amount of CuCO_3_/Cu_2_CO_3_(OH)_2_ in the surfaces of microcapsules. The group of 40%: 30%: 30% exhibited a relatively more uniform distribution of elements.

**FIGURE 4:**
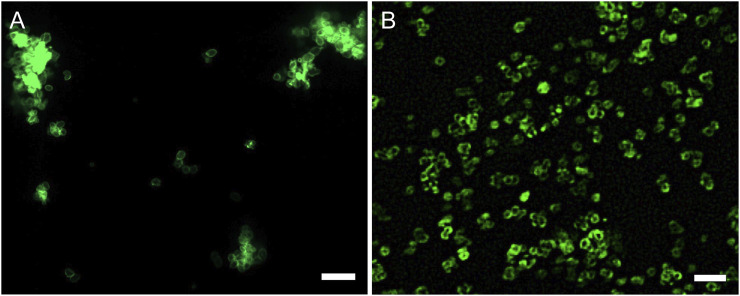
Micrographs of **(A)** FITC-labeled PAH coated at 3rd bilayer of CaCuMg-PEM-Col and **(B)** FITC-labeled collagen coated at the outmost layer (scale bar = 50 μm).

Fluorescence microscope imaging was applied to further visualize the adsorbed PEM and collagen on CaCuMg-CO_3_ crystals. As shown in Figure 4A, FITC-PAH was uniformly coated on the particles, forming a thin green layer along the particle surfaces. Similarly, the deposition of the FITC-collagen layer was also successfully observed on the surface of the encapsulated particles (Figure 4B). Interestingly, it was found that the CaCuMg-PEM-Col particles exhibited better dispersion than the CaCuMg-PEM particles after further adsorption of collagen. This is consistent with the measurement of the zeta potential. Since the adsorption of collagen leads to a higher negative charge distribution on the surface of the particles, the repulsion between the negatively charged particles is enhanced.

### 3.3 Ion release behavior of CaCuMg-PEM-Col microcapsules

**FIGURE 5:**
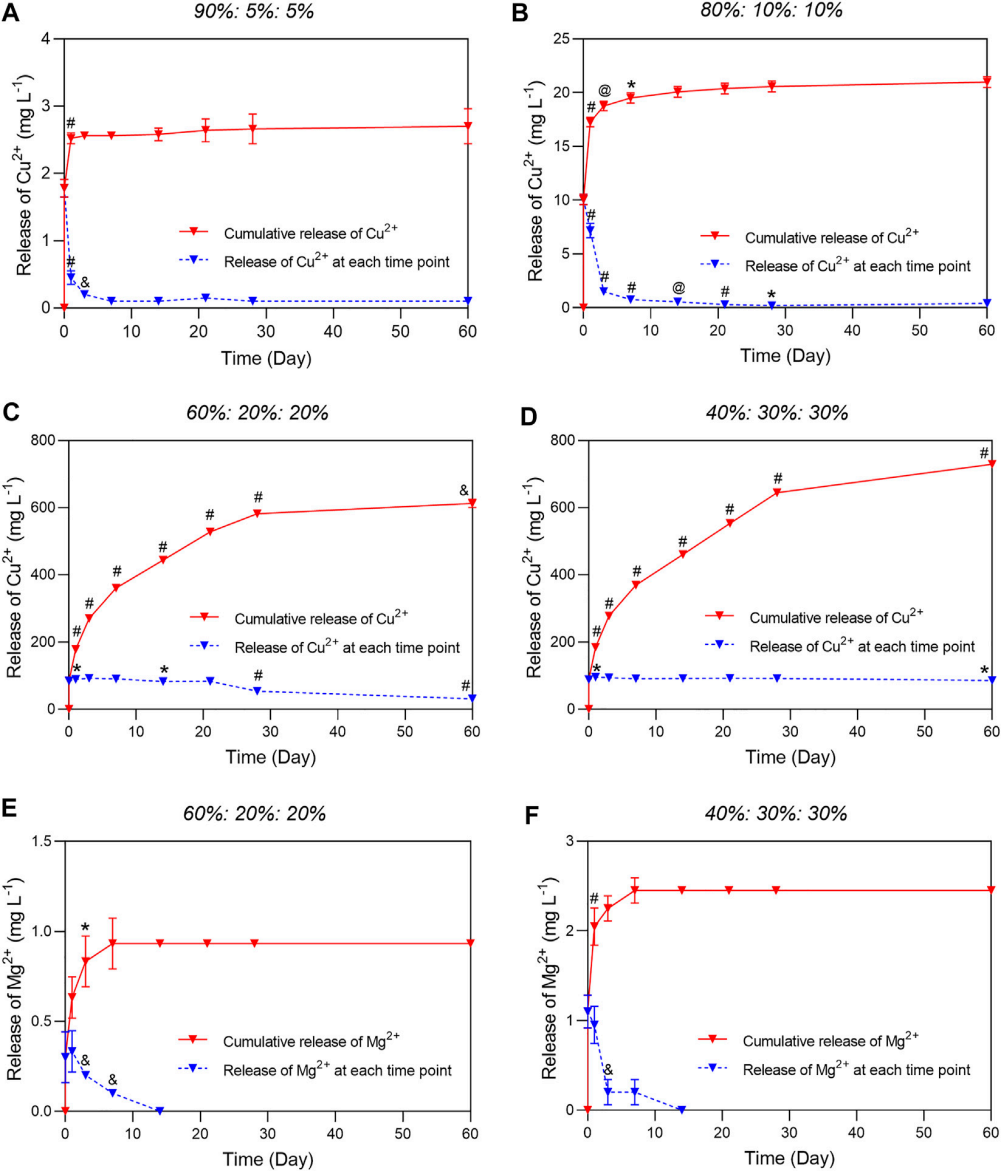
Ions release profile of CaCuMg-PEM-Col microcapsules determined by ICP-OES. The concentration of Cu^2+^ released from microcapsules prepared with different ratios of Ca^2+^, Cu^2+^, Mg^2+^: **(A)** *90%: 5%: 5%*, **(B)** *80%: 10%: 10%*, **(C)** *60%: 20%: 20%*, **(D)** *40%: 30%: 30%.* The release behavior of Mg^2+^ in **(E)** group of *60%: 20%: 20%* microcapsules and **(F)** that of *40%: 30%: 30%* microcapsules. (Mean ± SD, *n* = 5; Paired *t*-test, *p < 0.05, ^&^ *p* < 0.01, ^@^ *p* < 0.001, ^#^ *p* < 0.0001, indicated that there was a significant difference of released ions content between the two groups).

The release kinetics of the target bioactive cations Cu^2+^ and Mg^2+^ are of particular importance as a sudden increase in concentration due to excessive release of these cations will result in bone loss, irregular crosslinking or excessive cytotoxicity (Wong et al., 2014; Wang et al., 2016). Therefore, the stability and controllability of ion release kinetics during the long-term bone regeneration process is crucial. In this study, we investigated and compared the ion release behavior of the microcapsules prepared with different salt ratios *via* ICP-OES measurements. The two groups with higher Ca^2+^-content showed a burst release of Cu^2+^ in the first hours and 3 days, then followed by a very slow and incomplete release until day 60 (Figures 5A,B). While the two groups with increased Cu^2+^ and Mg^2+^ content showed stable release throughout the analyzed period without initial burst release (Figures 5C,D). Notably, the Mg^2+^ in the first two groups were below the detection limits of the ICP-OES. But in the groups of 60%: 20%: 20% and 40%: 30%: 30%, trace Mg^2+^ was slowly released in the first 7 days (Figures 5E,F). The amount of target bioactive ions Cu^2+^ and Mg^2+^ released from the capsules differed dramatically between the high and low Ca^2+^-content groups. The cumulative release profile of Ca^2+^ also showed an initial burst release in groups of 95%: 5%: 5% and 80%: 10%: 10% (Supplementary Figure S2). Interestingly, the concentration of Ca^2+^ decreased as the release process prolonged, whereas the Ca^2+^ release of the other two groups was stable and slow. When the Ca^2+^ content was replaced by increased Cu and Mg content, CaMgCO_3_ crystallizes in the nucleation region was surrounded by relatively unstable basic Cu and Mg(OH)_2_/MgCO_3_. In particular, the MgCO_3_ is destabilized by hydration to Mg(OH)_2_. This reaction cascade dynamically determines the surface morphology of the particles and the release behavior. The higher the Cu content the easier the Cu^2+^ can be released (Figures 5C,D). The unstable surface portion of the Cu/Mg was easily released within the first 7 days. As the dissolution front approaches the calcite core, the release was more limited by the dissolution of the calcite, especially for the Mg. This could explain the relatively short continuous release of Mg measured by ICP (Figures 5E,F). The release curve of Ca^2+^ also confirms the above mentioned mechanisms. After the release of unstable vaterite from the surface, the release rate decreased significantly for a short time, and then rose slowly because of the slow dissolution of calcite core (Supplementary Figure S2). The large amount of Mg^2+^ was increasingly co-crystalized in nucleation region of calcite or dolomite crystals which limited the dissolution process.

### 3.4 *In vitro* cytotoxicity

**FIGURE 6:**
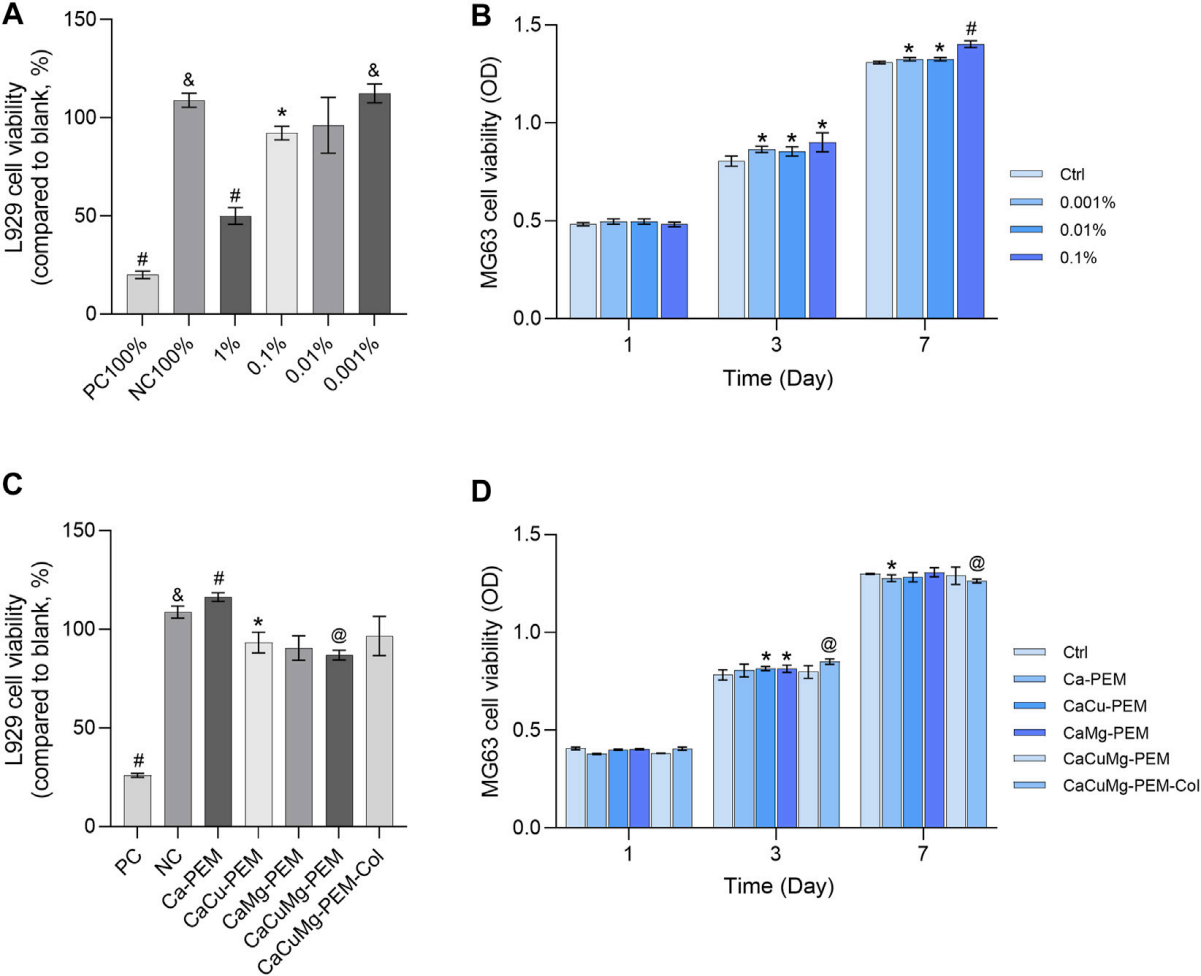
*In vitro* cytotoxicity of control samples (PC, positive control; NC, negative control) and **(A)** extract of CaCuMg-PEM-Col in a series of concentrations, and **(C)** extract of Ca-PEM, CaCu-PEM, CaMg-PEM, CaCuMg-PEM and CaCuMg-PEM-Col microcapsules towards L929 cells. Cell viability of osteoblast-like MG63 cells cultured with extract of microcapsules determined by CCK-8 assay at 1, 3, 7, 10 and 14 days: **(B)** culture medium supplemented with extract of CaCuMg-PEM-Col microcapsules in different concentrations; **(D)** culture medium supplemented with 0.1% extracts of Ca-PEM, CaCu-PEM, CaMg-PEM, CaCuMg-PEM and CaCuMg-PEM-Col microcapsules. (mean ± SD, *n* = 6; Paired *t*-test, *p < 0.05, ^&^ *p* < 0.01, ^@^ *p* < 0.001, ^#^ *p* < 0.0001, indicated that there was a significant difference of cell viability in comparison to blank control).

In addition to achieve the sustained release function as an encapsulation system, PEM and collagen were also used to mimic bone tissue ECM to promote osteogenic differentiation *via* modifying different surface properties which in turn stimulating numerous interfacial interactions with the cells adsorbed to the coated surfaces (Brito Barrera et al., 2020; Sankar et al., 2021). Collagen is an indispensable matrix protein that stimulates various signaling pathways such as cell adhesion, mobility, wound healing, proliferation, and an important process to complete bone regeneration, named ECM remodeling (Zhang et al., 2018). Other physicochemical properties of the surface are also decisive for proper bone regeneration, such as surface roughness, hardness, electrical charge and charge density (Bharadwaz and Jayasuriya, 2020). The PEM provides a highly tunable surface fulfilling these demands. The negative charge of the coated surface attracts cells and makes them easy to adhere; in addition, cell adhesion and the necessary cell motility are more active on harder surfaces than on softer ones (Sahebalzamani et al., 2022). Therefore, a PEM-Col surface has been chosen for proof of this strategy. In this study, mouse derived L929 fibroblasts were used to evaluate the cytotoxicity of CaCuMg-PEM-Col microcapsules. The cell viability assay has been performed *via* an extraction test on L929 and evaluated by CCK-8 assay. According to the ISO standard, metabolic activity below 70% is considered to be cytotoxic. As shown in Figure 6A, the microcapsules showed a decrease in cytotoxicity with decreasing extract concentration, while the viability of fibroblasts was below 70% only when the extract concentration was higher than 1%. According to the results of ICP measurements as shown in Figure 5, the concentration of Cu^2+^ in 1% extract was 15.63 µM and the concentration of Mg^2+^ is 4.17 µM. These measurements formed the basis for determining a suitable dosage range of the microcapsules. To exclude the bias due to PEM, collagen and their probable degradation products, the extracts of five types of microcapsules have been applied for the cytotoxic evaluation, including Ca-PEM, CaCu-PEM, CaMg-PEM, CaCuMg-PEM and CaCuMg-PEM-Col. All the extracts did not exhibit any significant cytotoxicity on L929 cells (Figure 6C), suggesting good biocompatibility of the microcapsules. It was found that the cells treated with CaCuMg-PEM-Col extract showed higher viability compared to CaCuMg-PEM without a collagen layer, which might be a result of slight desorption of collagen from the surface of the microcapsules. The beneficial effects from the collagen layer might also reduce the toxicity resulting from Mg^2+^ and Cu^2+^ ions within certain concentration threshold (Cu^2+^: 15.63 µM; Mg^2+^: 4.17 µM).

Main task of this trimetal microcapsules is to stimulate the bone regeneration *via* controlled release of these bioactive cations. Therefore, cell proliferation of osteoblast-like MG63 cells was also carried out by using CCK-8 assay on the cell cultures at 1st, 3rd, and 7th days. Results in Figure 6B showed that cell viability on the 1st day was not markedly affected in any condition of microcapsules extracts (0.001%, 0.01%, and 0.1%) because the cells need sufficient time to recover after the passaging and transfer into the test-cultures. On the 3rd day, cells viability higher than 70% was observed for the groups treated with ionic extracts, all of which were higher than those of the blank controls. The cell viability of the group of 0.1% of microcapsule extract was obviously higher than other groups on the 7^th^ day. A similar trend was observed with cell viability measured by addition of Ca-PEM, CaCu-PEM, CaMg-PEM, CaCuMg-PEM and CaCuMg-PEM-Col capsules (Figure 6D). Results revealed that the cell proliferation ability of the microcapsule groups was similar to or higher than that of blank control. And the cells in the CaCuMg-PEM-Col group showed the highest viability on the 3rd and 7th day. These results indicate that metal ions and CaCuMg-PEM-Col microcapsules did not induce any cytotoxic effect but promoted osteoblast proliferation in conjunction with type I collagen.

### 3.5 Osteogenic differentiation of osteoblast-like MG63 cells *in vitro*

**FIGURE 7:**
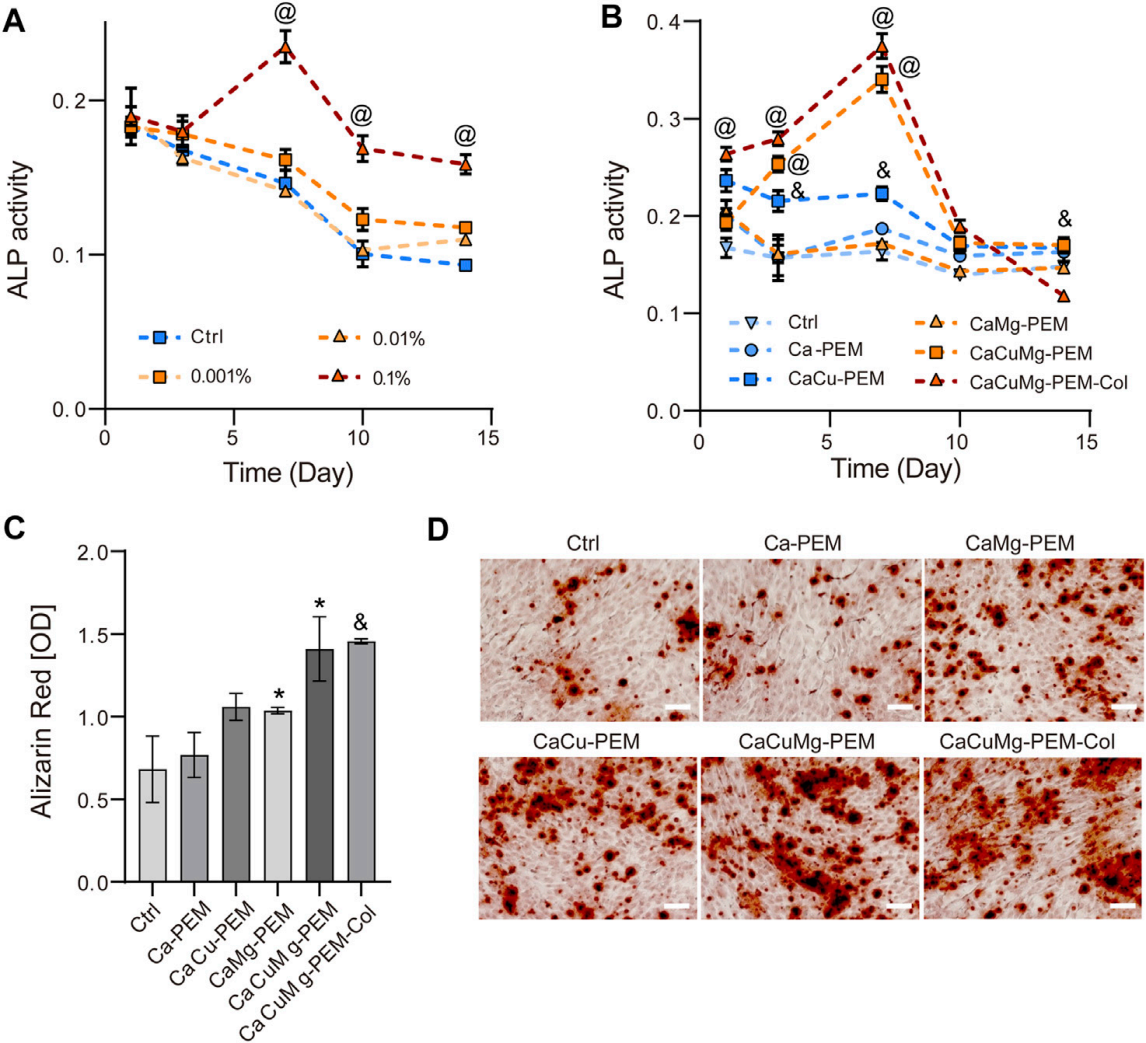
ALP activity was evaluated after 1, 3, 7, 10, 14 days with colorimetric method with pNPP as a substrate for ALP: **(A)** MG63 cells cultured with osteogenic medium supplemented with extract of CaCuMg-PEM-Col microcapsules in different concentrations; **(B)** MG63 cells cultured with osteogenic medium supplemented with 1% extracts of Ca-PEM, CaCu-PEM, CaMg-PEM, CaCuMg-PEM and CaCuMg-PEM-Col microcapsules. Osteogenic differentiation was revealed by **(D)** Alizarin Red staining (scale bar = 100 μm) and **(C)** quantification at day 21. (*n* = 3; Paired *t*-test, *p < 0.05, ^&^ *p* < 0.01, ^@^ *p* < 0.001).

Bone regeneration mainly includes proliferation and differentiation of osteoblasts. The previous viability measurements confirmed the positive effects of CaCuMg-PEM-Col microcapsules on cell proliferation. Therefore, the impacts on osteogenic differentiation were further investigated. ALP is an early marker of osteoblast differentiation which is an important indicator for bone formation and mineralization as well as for the bone regeneration progress (Hu & Olsen, 2016). The cellular ALP activity of MG63 cells treated with different microcapsule extracts were monitored on the 1st, 3rd, 7th, 10th and 14th days. As shown in Figure 7A, on the 1st and 3rd day, ALP activity of cells treated with the extract of CaCuMg-PEM-Col microcapsules (0.001%, 0.01%, and 0.1%) were at a similar level as the blank control. Cellular ALP activity of the group of 0.1% reached peak on the 7th day, which was significantly higher than groups of 0.001%, 0.01% and ctrl. From day 7 onwards, decrease in ALP activity was observed under all conditions. Nevertheless, ALP activity of cells treated with 0.1% of microcapsule extracts was still significantly higher than other treated groups and control group. Therefore, 0.1% was adopted as the concentration used for the subsequent investigation (Figure 7B). On the 1st day, cells treated with osteogenic medium supplemented with sample extracts including Ca-PEM, CaCu-PEM, CaMg-PEM, CaCuMg-PEM and CaCuMg-PEM-Col showed higher ALP activity than control group which was only treated with osteogenic medium. On days 3 and 7, the levels and trends of ALP activity in the Ca-PEM and CaMg-PEM groups were the same as those in the blank control group, first increasing and then slowly decreasing. However, there was no significant difference in the expression levels between groups. The group of CaCu-PEM showed a similar trend but significantly higher ALP activity at these time points. Notably, the levels of ALP activity in the CaCuMg-PEM and CaCuMg-PEM-Col groups continued to increase over time, reaching a peak on day 7, in which the ALP activity in these two groups was significantly higher than other groups, and gradually decreased thereafter to levels close to those of the other groups. This indicates the significant synergistic regulation of Cu^2+^ and Mg^2+^ and collagen in osteoblasts differentiation. Interestingly, the ALP activity of the CaCuMg-PEM-Col group also showed the largest decrease among all groups after exhibiting the highest levels on days 1, 3, 7, and 10. It is suggested that CaCuMg-PEM microcapsules further functionalized with collagen could better mimic ECM, thus providing a more suitable microenvironment for bone regeneration and promoting the proliferation and differentiation of osteoblasts. ALP activity downregulated by CaCuMg-PEM-Col at day 14 indicated that the initiation of ECM mineralization completed earlier than other groups.

The effect of CaCuMg-PEM-Col on late differentiation (matrix mineralization) was investigated after 21 days of culture *via* Alizarin Red staining and quantification. As shown in Figure 7D, stronger Alizarin Red staining was observed on CaCuMg-PEM and CaCuMg-PEM-Col as compared to CaCu-PEM, CaMg-PEM, and blank control. Similarly, the result of quantification shows that mineralization on CaCu-PEM (*p* < 0.05), CaCuMg-PEM (*p* < 0.05) and CaCuMg-PEM-Col (*p* < 0.01) were significantly higher than the control group, suggesting that the microcapsules have a positive influence on osteoblast mineralization (Figure 7C).

### 3.6 Expression of osteogenesis and angiogenesis-related genes

**FIGURE 8:**
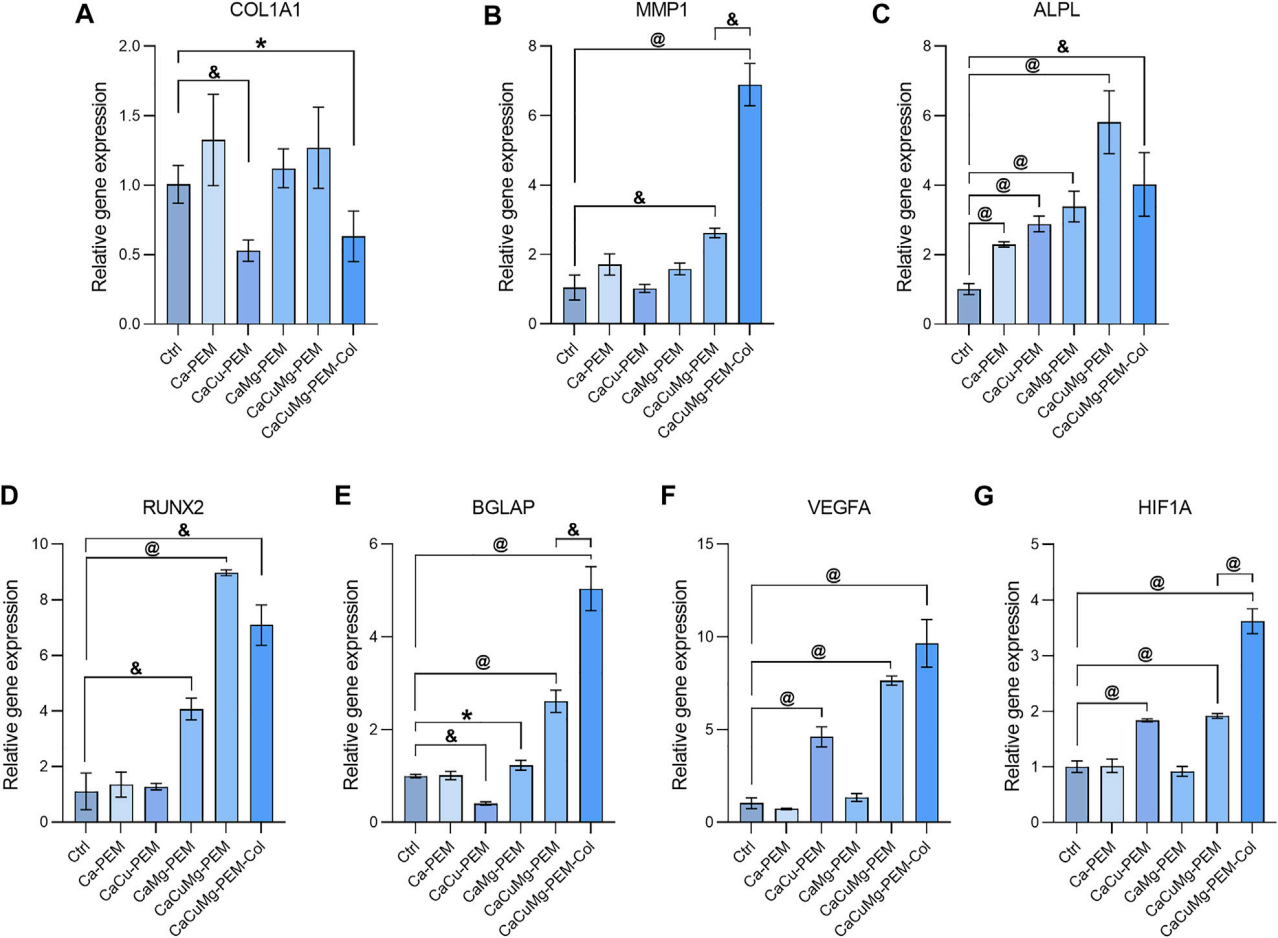
Relative gene expression of **(A**–**E)** osteogenic markers and **(F**,**G)** angiogenesis markers at 14 days analyzed *via* qRT-PCR (*n* = 3; Paired *t*-test, *p < 0.05, ^&^ *p* < 0.01, ^@^ *p* < 0.001).

As discussed previously, the bone regeneration process can be briefly divided into three periods: proliferation, ECM maturation and mineralization. Two transition points of gene expression of certain marker genes controlling cell proliferation and differentiation have been reported (Stein et al., 1990). The first transition occurs after completion of proliferation and initiation of ECM, when ALP and collagen are upregulated. The second transition takes place at the initiating of ECM mineralization. In this study, we investigated the gene expression related to osteogenesis and angiogenesis of MG63 at day 14 *via* real-time PCR (Lynch et al., 1995). As shown in Figure 8A, the COL1A1 expression of CaCu-PEM and CaCuMg-PEM-Col were significantly lower than blank control (osteogenic medium). This might result by the ECM-mimicking effect from the PEM and PEM-Col capsules. Reasonably, when compared to the blank control the matrix metalloproteinase-1 (MMP1) gene expression was markedly higher in the CaCuMg-PEM group (*p* < 0.01) and CaCuMg-PEM-Col group (*p* < 0.001) due to the presence of collagen (Figure 8B). All groups of microcapsules (Ca-PEM, CaCu-PEM, CaMg-PEM, CaCuMg-PEM, and CaCuMg-PEM-Col) showed significantly higher expression of ALPL in comparison to the blank control (osteogenic medium), among which the expression in the CaCuMg-PEM group was the highest, followed by the CaCuMg-PEM-Col (Figure 8C). The expression of RUNX2 in groups of CaMg-PEM, CaCuMg-PEM, and CaCuMg-PEM-Col were significantly higher than other groups (Figure 8D). The expression pattern of osteocalcin (BGLAP) was roughly similar to that of MMP1, showed the highest level in the CaCuMg-PEM-Col group, followed by the CaCuMg-PEM group (Figure 8E).

During osteogenic differentiation, (pre-) osteoblasts play a central role in communication with endothelial cells to ensure the temporal and spatial coupling of osteogenesis and angiogenesis *via* the regulation of gene expressions involved in angiogenesis. Interestingly, high expression of VEGFA and HIF1A were found in osteoblasts conditioned with CaCu-PEM, CaCuMg-PEM, and CaCuMg-PEM-Col extracts as shown in Figures 8F,G, suggesting that copper plays a more important role in the promotion of angiogenesis. Notably, we found significant synergistic effects of copper and magnesium, as well as the addition of type I collagen, on angiogenesis.

## 4 Conclusion

Bioactive metal ions including Ca^2+^, Cu^2+^ and Mg^2+^ play a predominant role in the process of bone regeneration, which are recognized as an alternative for bone growth factor-based therapeutics. However, a delivery system with high stability and loading capacity of multiple metal ions, and controlled release kinetics is currently highly required. In this study, the vaterite-calcite CaCO_3_ particles were effectively loaded with Cu^2+^ and Mg^2+^, then coated with PEM to improve the crystal stability for better sustained release behavior, and further successfully functionalized with collagen to mimic bone tissue ECM. Ca^2+^, Cu^2+^ and Mg^2+^ could sustainably release from the microcapsules and induce a proper bone regeneration microenvironment, regulating the osteoblasts proliferation and differentiation, promoting the ECM maturation and mineralization. It was shown that both osteogenesis and angiogenesis-related gene expressions were upregulated. Therefore, CaCuMg-PEM-Col microcapsules present a type of bioactive metal ion encapsulation and delivery system for the functionalization of bone graft materials. The presented strategy of combining multi metal ions with biocompatible PEM and collagen provides new inspiration and important prospects for bone tissue engineering.

## Data Availability

The original contributions presented in the study are included in the article/Supplementary Materials, further inquiries can be directed to the corresponding authors.
