# Supplementary material for: Encapsulated vaterite-calcite CaCO3 particles loaded with Mg2+ and Cu2+ ions with sustained release promoting osteogenesis and angiogenesis
Source: Front Bioeng Biotechnol. 2022 Aug 11;10:983988. doi: 10.3389/fbioe.2022.983988 (PMC9403055; doi:10.3389/fbioe.2022.983988)
Supplement: Supplementary file 1 [file DataSheet1.docx]

Supplementary Material

Encapsulated vaterite-calcite CaCO_3_ particles loaded with Mg^2+^ and Cu^2+^ ions with sustained release promoting osteogenesis and angiogenesis

Lu Fan^1,2^, Fabian Körte^1^, Alexander Rudt^3^, Ole Jung^4^, Claus Burkhardt^1^, Mike Barbeck^4^*, Xin Xiong^1^*

^1^ NMI Natural and Medical Sciences Institute at the University of Tübingen, Markwiesenstr. 55, 72770 Reutlingen, Germany

^2^ Experimental Medicine, Faculty of Medicine, University of Tübingen, Geschwister-Scholl-Platz, 72074 Tübingen, Germany

^3^ Faculty of Applied Chemistry, Reutlingen University, Alteburgstr. 150, 72762 Reutlingen, Germany

^4^ Medical Center of Rostock University, Schillingallee 35, 18057 Rostock, Germany

* Correspondence:
Mike Barbeck
mike.barbeck@ med.uni-rostock.de
Xin Xiong
xin.xiong@nmi.de


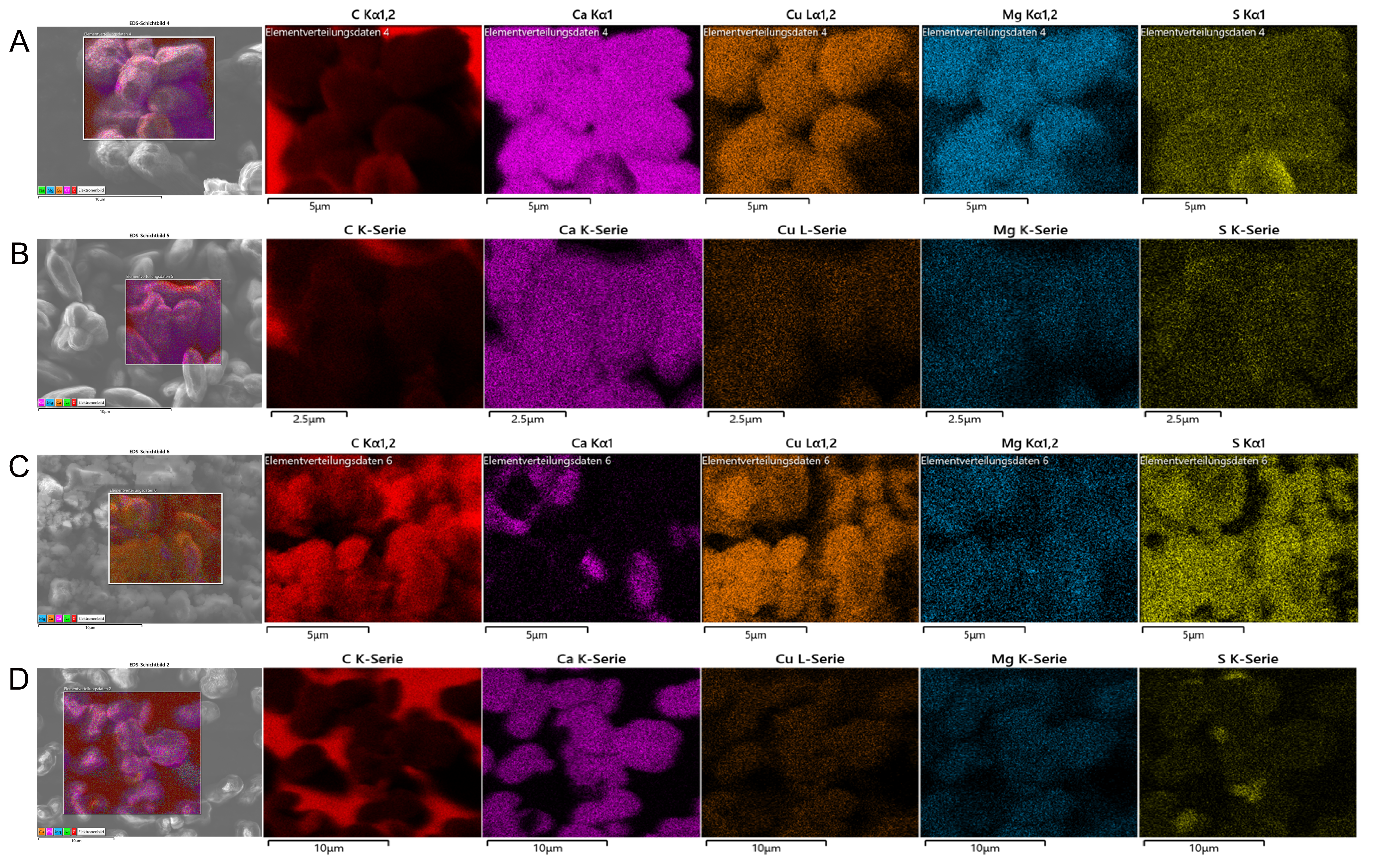
 **Figure S1.** SEM-EDS-Mapping of CaCuMg-PEM-Col microcapsules prepared with different ratios of salts: (A) Ca^2+^: Cu^2+^: Mg^2+^ = *90%: 5%: 5%*, (B) Ca^2+^: Cu^2+^: Mg^2+^ = *80%: 10%: 10%*, (C) Ca^2+^: Cu^2+^: Mg^2+^ = *60%: 20%: 20%*, (D) Ca^2+^: Cu^2+^: Mg^2+^ = *40%: 30%: 30%*.


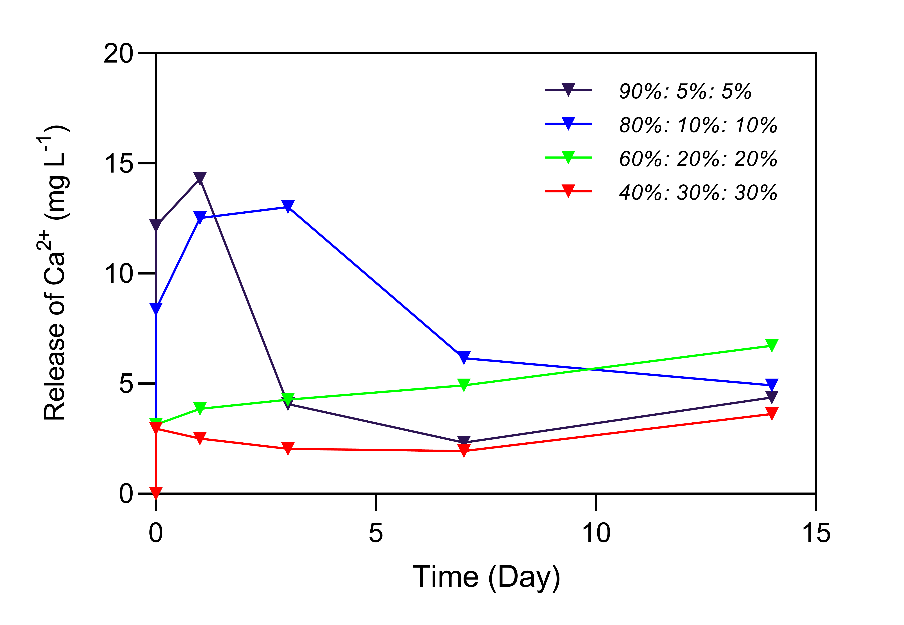


**Figure S2.** The cumulative release profile of Ca^2+^ from microcapsules determined by ICP-OES.

**Table S1.** The fitting of thickness and mass after coating process and overnight incubation in ddH_2_O via QSense_Dfinder. The coating processes of [PSS/PAH]_5_PSS-Col and [PSS/PAH]_5_-Col were performed on 3 QCM sensors (*S1, S2* and *S3*) respectively. R3 and R5 refer to the readout from overtone 3 and 5.

|  | | Coating process | | | | | |  | | After overnight incubation | | | |
| --- | --- | --- | --- | --- | --- | --- | --- | --- | --- | --- | --- | --- | --- |
|  | |  |  | *S1* | *S2* | *S3* | |  | | *S1* | *S2* | *S3* | |
| [PSS/PAH]_5_PSS-Col | | Thickness (nm) | R3 | 63.93 | 55.96 | 58.01 | |  | | -16.16 | -13.15 | -13.94 | |
|  |  |  | R5 | 58.83 | 55.4 | 52.74 | |  | | -15.08 | -13.46 | -12.63 | |
|  |  | Mass (ng cm^-2^) | R3 | 6393.24 | 5801.19 | 5596.39 | |  | | -1616.38 | -1314.58 | -1616.38 | |
|  |  |  | R5 | 5882.88 | 5540.42 | 5273.55 | |  | | -1507.67 | -1263.38 | -1348.77 | |
|  | |  |  |  |  |  | |  | |  |  |  | |
| [PSS/PAH]_5_-Col | | Thickness (nm) | R3 | 87.62 | 74.82 | 70.61 | |  | | -0.5 | -3.69 | -16.1 | |
|  |  |  | R5 | 77.58 | 65.69 | 63.68 | |  | | 0.98 | -9.03 | -6.17 | |
|  |  | Mass (ng cm^-2^) | R3 | 8762.08 | 7482.02 | 7061.45 | |  | | 53.52 | -369.35 | -1610.05 | |
|  |  |  | R5 | 7758.19 | 6569.31 | 6368.22 | |  | | 97.99 | -616.55 | -903.33 | |

**Table S2.** The element composition ratio obtained from SEM-EDS-Spectra.

|  | C | O | Na | Ca | Cu | Mg | S | Total |
| --- | --- | --- | --- | --- | --- | --- | --- | --- |
| Ca^2+^: Cu^2+^: Mg^2+^ = *90%: 5%: 5%* | 32.45 | 31.83 | 0.29 | 33.28 | 1.04 | 0.45 | 0.66 | 100 |
| Ca^2+^: Cu^2+^: Mg^2+^ = *80%: 10%: 10%* | 25.26 | 34.56 | 0.25 | 36.19 | 1.88 | 0.98 | 0.87 | 100 |
| Ca^2+^: Cu^2+^: Mg^2+^ = *60%: 20%: 20%* | 67.49 | 6.79 | 0 | 5.8 | 13.05 | 0.09 | 6.79 | 100 |
| Ca^2+^: Cu^2+^: Mg^2+^ = *40%: 30%: 30%* | 15.15 | 41.81 | 0.39 | 40.26 | 1.36 | 0.64 | 0.38 | 100 |

**Table S3.** Gene primers of reverse-transcription and real-time PCR.

| Gene icon | Gene name | Assay ID (Thermo Fisher Scientific) |
| --- | --- | --- |
| COL1A1 | Collagen type I alpha 1 | Hs00164004_m1 |
| MMP1 | Matrix metallopeptidase 1 | Hs00899658_m1 |
| ALPL | Alkaline phosphatase | Hs01029144_m1 |
| RUNX2 | Runt related transcription factor 2 | Hs01047973_m1 |
| BGLAP | Bone gamma-carboxyglutamate protein | Hs01587814_g1 |
| VEGFA | Vascular endothelial growth factor A | Hs00900055_m1 |
| HIF1A | Hypoxia inducible factor 1 alpha subunit | Hs00153153_m1 |
| GAPDH | Glyceraldehyde-3-phosphate dehydrogenase | Hs99999905_m1 |
